# The Epidemiological Study of Coxsackievirus A6 revealing Hand, Foot and Mouth Disease Epidemic patterns in Guangdong, China

**DOI:** 10.1038/srep10550

**Published:** 2015-05-21

**Authors:** Hanri Zeng, Jing Lu, Huanying Zheng, Lina Yi, Xue Guo, Leng Liu, Shannon Rutherford, Limei Sun, Xiaohua Tan, Hui Li, Changwen Ke, Jinyan Lin

**Affiliations:** 1 Key Laboratory for Repository and Application of Pathogenic Microbiology, Research Center for Pathogens Detection Technology of Emerging Infectious Diseases, Guangdong Provincial Center for Disease Control and Prevention, Guangzhou, China; 2 Guangdong Provincial Institute of Public Health, Guangdong Provincial Center for Disease Control and Prevention, Guangzhou, China; 3 WHO Collaborating Centre for Surveillance, Research and Training of Emerging Infectious Diseases, Guangzhou, 511430, China; 4 Centre for Environment and Population Health, Nathan campus, Griffith University, 170 Kessels Road, Nathan Brisbane, Queensland 4111, Australia

## Abstract

Enterovirus A71 (EVA71) and Coxsackievirus A16 (CVA16) are regarded as the two major causative pathogens in hand, foot and mouth disease (HFMD) epidemics. However, CVA6, previously largely ignored, became the predominant pathogen in China in 2013. In this study, we describe the epidemiological trendsofCVA6 during the annual HFMD outbreaks from 2008 to 2013 in Guangdong, China. The study results show that CVA6 has been one of three major causative agents of HFMD epidemics since 2009. The periodic rotation and dominance of the three pathogens, EVA71, CVA16 and CVA6, may have contributed to the continuously increasing HFMD epidemics. Moreover, phylogenetic analysis of the VP1 gene shows that major circulating CVA6 strains collected from 2009 to 2013 are distinct from the earlier strains collected before 2009. In conclusion, the discovery from this research investigating epidemiological trends of CVA6 from 2008 to 2013 explains the possible pattern of the continuous HFMD epidemic in China. The etiological change pattern also highlights the need for improvement for pathogen surveillance and vaccine strategies for HFMD control in China.

Hand-foot-mouth disease (HFMD) is a common childhood disease caused by human *enterovirus A* species. HFMD is typically characterized by fever, sore throat, general malaise and vesicular rash on the hands and feet as well as exanthema on oral mucosa and tongue^1^. In the last few decades, HFMD has been a very common infection in children in the Asia-Pacific region^2^,^3^,^4^,^5^,^6^, and sporadic outbreaks have also been described in Europe and North America in recent years^7^,^8^. In particular, in the last ten years mainland China is the country most affected by hand, foot and mouth disease (HFMD) with largest number of HFMD cases and associated deaths ( http://www.nhfpc.gov.cn/, in Chinese).

Two types of *enterovirus A* species, enterovirus A71 (EVA71) and coxsackievirus A16 (CVA16) are regarded as the major causative agents for HFMD^9^,^10^,^11^. Compared to other enteroviruses, EVA71 is more often associated with neurological diseases such as meningitis, encephalitis, monoplegia, acute flaccid paralysis and sometimes death especially among children under 5 years old 12,13.The first EVA71 vaccine has reached phase 3 clinical testing in China. The vaccine could give 90% protection against clinical EVA71-associated hand, foot and mouth disease and 80.4% protection against EVA71-associated disease, including neurological complications, for at least 12 months^14^,^15^. While, another serotype, CVA6, has been increasingly associated with HFMD cases or outbreaks globally in the last 5-6 years. For example in Finland, CVA6 was identified as the primary virus serotype associated with HFMD during a nationwide outbreak in 2008^7^ and a prospective observational study conducted in France indicated that CVA6 accounted for 26% of HFMD or herpangina cases during an outbreak in 2010. In Spain, CVA6 replaced CVA16 to be the predominant pathogen during HFMD outbreaks in 2011 and 2012^16^. Similarly, outbreaks of HFMD caused by CVA6 have also been frequently reported in the Asia-Pacific region including Singapore in 2008^17^, Taiwan in 2010^18^, Japan in 2011^19^ and India in 2012^20^.

In mainland China, HFMD has been listed as a notifiable disease since 2008. Two serotypes of enterovirusEVA71 and CVA16were regarded as the two major causes for the repeated national HFMD outbreaks from 2008 to 2012^21^. However, CVA6 which had been largely ignored, replaced EVA71 and CVA16 as the predominant pathogen for the HFMD epidemic in Guangdong, China in 2013^22^. Similar trends were also reported in Jilin Province^23^ and Beijing city^24^ in northern China in 2013. Together, these observations provide strong evidence of CVA6 infections as a new and important cause of HFMD. Compared to EVA71, our understanding of the epidemiology of CVA6 is limited by a relative lack of continuous surveillance data. In this study, we analyzed the CVA6 prevalence from 2008 to 2013 in Guangdong province. Phylogenetic analysis on VP1 genes of 163 CVA6 strains collected from 2004 to 2013 was also performed to describe the genetic characteristics of CVA6 in China.

## Results

### Clinical and epidemiological data

In Guangdong, positive samples were collected from 4971 patients between 2008-13 with case ages ranging from 1 month to 40 years. The median age of CVA6 positive cases was 17 months (range of 1 month to 11 years), which was similar to the median age for EVA71 and CVA16 positive cases (Table 1). Similarly, no significant differences were observed among different enterovirus serotypes in terms of gender distribution (Table 1).

HFMD cases were classified into mild and severe based on the diagnosis guidance from the National Health and Family Planning Commission of China^25^. Among 4971enterovirus positive HFMD patients, 598 (12%) were diagnosed with severe HFMD. EVA71 was significantly associated with a higher risk of severe HFMD (450 in 1560, 28.8%) when compared to CVA16 (32 in 997, 3.3%) or CVA6 (37 in 1026, 3.6%). Similar to the CVA16 virus, the majority of CVA6 cases presented mild symptom with only 3.6% of CVA6 positive cases diagnosed as severe cases.

### Prevalent enterovirus genotypes

**Figure 1:**
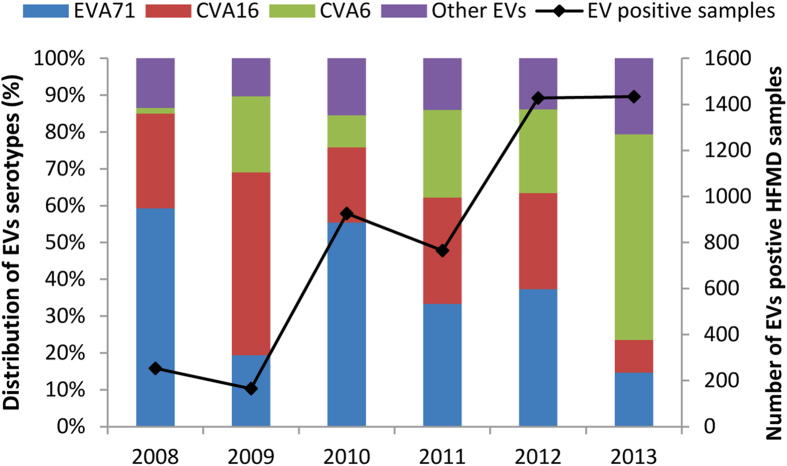
Enterovirus assoicated HFMD cases and enterovirus distribution in Guangdong Province, China, from 2008 to 2013. The continuous line describes the total number of HFMD cases collected from 28 sentinel hospitals each year in 21 cities of Guangdong; the histogram shows the percentage of EVA71, CVA16, CVA6 and other untyped enterovirus (EVs) isolates in EV positive samples.

The incidence of HFMD has gradually increased in Guangdong province. According to the provincial web-based surveillance system, there are 48917, 93067, 226622, 274006, 330621, and 358068 HFMD cases reported in 2008, 2009, 2010, 2011, 2012 and 2013, respectively. Corresponding to these figures are large observed increases in enterovirus positive samples collected from sentinel hospitals (Fig. 1).To visualize the epidemiological trends of CVA6, the annual distributions of enterovirus serotypes from 2008 to 2013 are presented (Fig. 1). Consistent with previous reports, EVA71 and CVA16 represented two major circulating serotypes of enterovirus in 2008^26^.With the exception of 2009, from 2008 to 2012, EVA71 was the most predominant enterovirus and represented more than 30% of enterovirus positive samples. CVA16 accounted for more than 20% of enterovirus positive cases from 2008 to 2012, and showed the highest epidemic activity in 2009 where it accounted for 49.7% of all detected cases. Intriguingly, CVA6 which was rarely detected (1.6%) in the earliest epidemic year of 2008, accounted for 20.6% enterovirus positive HFMD cases in 2009, 23.8% of cases in 2011 and 22.8% of cases in 2012. In the 2013 outbreak, CVA6 replaced EVA71 and CVA16 as the major (55.8%) causative pathogen. Across all years, CVA6 represented one of the major circulating enterovirus (24% of all enterovirus positive cases) from 2008 to 2013 in Guangdong. Interestingly, the three types of enterovirus represented about 80% of enterovirus positive HFMD cases each year (Fig. 1). This proportion was quite stable in the last six years despite the variable contribution of each serotype from year to year.

### Phylogenetic analysis

CVA6 virus transmission and evolution has rarely been assessed in China due to lack of long-term surveillance. In this study, phylogenetic analysis of CVA6 strains from 2008 to 2013 in Guangdong was performed and compared with reference strains from other regions in GenBank database. To better understand the genetic differences and virus evolution of CVA6 in Guangdong, we also included 9 CVA6 strains isolated from 2004 to 2007 from AFP patients into the analysis. Preliminary phylogenetic analysis of all CVA6 sequences was shown in supplemental Fig. 1. Representative strains from each year were selected to more concisely show the chronologic genetic diversity of CVA6 strains (Fig. 1).

CVA6 strains were classified into eight major sub-genotypes (denoted A-G) based on the full length of the VP1 gene. The prototype strain of CVA6 isolated in the USA in 1949 was the unique member of genotype G. The CVA6 strains isolated in Shandong Province in 1992 and 1996 represented the earliest CVA6 strains in mainland China. These two strains were segregated into distinct cluster E and cluster F suggesting there may be different transmission pathways for these early CVA6 strains in China. A great genetic distance was also identified among the six CVA6 strains isolated from 2004 to 2007 in Guangdong. Five Guangdong CVA6 strains isolated from 2004 to 2007 fell into cluster F and likely originated from the CVA6 strain JQ364886(SD/CHN/92) which was isolated in Shandong, China in 1992. The other four strains A448(GD/CHN07), A454(GD/CHN07), A442(GD/CHN06) and A453(GD/CHN07) represented the earliest strains in cluster D and were likely the origin of viral strains isolated from the HFMD epidemic during the 2008 to 2011 period in China.

**Figure 2:**
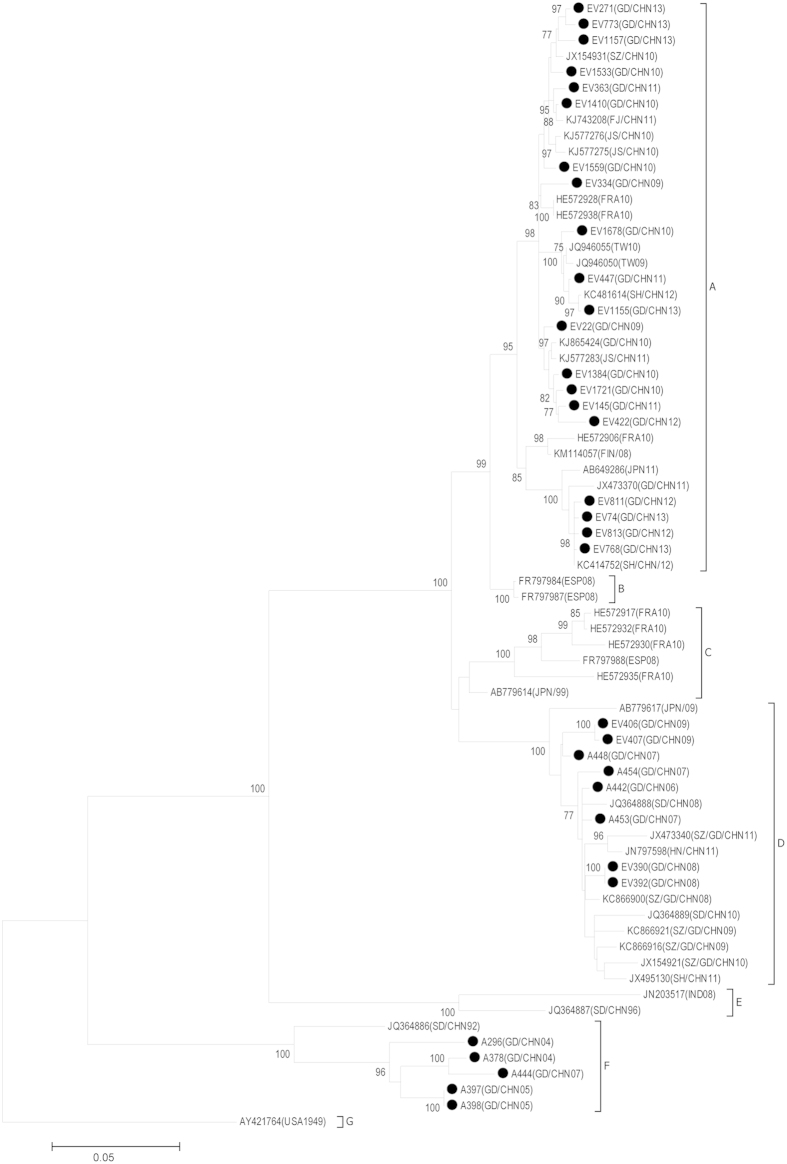
Phylogenetic analysis of coxsackievirus A6 viral capsid protein 1 nucleotide sequences (nt 2441 to 3355 according to Gdula strain AY421764) showing the relationships between CVA6 strains. Genbank accession numbers and location as well as year for virus isolation are included. Scale bar indicates branch distances; solid circles indicate strains sequenced in this study. Only bootstrap values of over 75% are shown.

CVA6 strains isolated in 2008 in China were uniquely grouped into cluster D. In contrast, viral strains isolated in China thereafter from 2009 to 2011 were dispersed into two different clusters (cluster A and cluster D). Over time, more and more CVA6 strains isolated in mainland China fell into cluster A suggesting viral strains in this cluster may present higher epidemic activity. As a result, almost all of the CVA6 strains isolated during the 2012 and 2013 HFMD epidemic in Guangdong, Shanghai, Jiangsu, Shandong, and Fujian were segregated into one major genetic cluster A (Fig. 2), and displayed a close genetic relationship with the 2008 Finland strain, 2009/2010 Taiwan strains, 2010 French strains and 2011 Japanese strains associated with HFMD outbreaks^19^,^27^,^28^,^29^.

## Discussion

CVA6, increasingly detected in some European countries since 2008, has been associated with the major cause of several HFMD outbreaks in Europe and Asia^7^,^16^,^17^,^18^,^19^,^28^,^29^,^30^,^31^,^32^. In mainland China, CVA6 was not a great concern until the outbreak of HFMD in 2013. In that outbreak, CVA6 accounted for more than 50% of all infections and replaced EVA71 to be the predominant pathogen in the HFMD epidemic^22^,^23^,^24^. Based on the surveillance on thousands of HFMD cases, the present study suggests that CVA6 infection is comparable to CVA16 infection and mainly leads to mild diseases during epidemic periods. However, detailed descriptions of severe cases were not available from the web-based surveillance system and therefore, the differences in severe syndrome caused by CVA6 infections and other enterovirus infections remain unclear. The most recent research suggests that meningitis rather than encephalitis was identified in CVA6 associated severe cases and the CVA6 associated severe cases showed higher fever and shorter fever duration when compared with the EVA71 manifestation^24^,^33^. In addition, HFMD patients with CA6 infection had more widespread skin lesions when compared with CVA16 or EVA71 infection^18^,^24^,^27^,^34^,^35^,^36^. CVA6 patients always have skin rashes beyond the typical sites for HFMD, including trunk, neck, face, and perioral area^18^,^27^,^34^,^35^. Desquamation of the palms and soles as well as nail abnormalities were also commonly found after the infection episode^18^,^24^,^37^.

Though several studies have been described the emergence and dominance of CVA6 in HFMD epidemic in mainland China^22^,^23^,^24^,^33^,^37^,^38^,^39^,^40^, there are at least two points needed to be further clarified. First, the circulation pattern of CVA6 during the long term HFMD epidemics in mainland China is still largely unknown. To date, most epidemiology studies of CVA6 in mainland China were limited in more recent years (2010-2013) 23,37,38,39 and only one study conducted by *He et al*. described the distributions and genetic characters of CVA6 in Shenzhen city from 2008 to 2012^40^. Second, all of phylogenetic analyses in these studies were based on the partial sequence of the VP1 gene. Previous results suggested CVA6 strains collected from HFMD epidemic from 2010 to 2013 in mainland China belonged to a single cluster except the study done by *He et al.*^40^. While, the caution should be applied that short VP1 sequences may be not sufficient to distinguish differences of CVA6 strains collected from different epidemic years.

In this study, we included full VP1 sequences data from HFMD surveillance from 2008 to 2013. Moreover, the CVA6 strains isolated from AFP patients from 2004 to 2007 were also included in this study to better elucidate the genetic variances of CVA6 circulating in Guangdong China. The study result shows that major circulating CVA6 strains collected from 2009 to 2013 are distinct from the earlier strains collected before 2009. The phylogenetic analysis shows the CVA6 strains circulating in 2012-2013 and the strains isolated in 2008 or earlier in China are segregated into different clusters. The cluster A in Fig. 2 represents the major circulating CVA6 strains in mainland China after 2008. In this cluster, the CVA6 strain (KM114057) isolated from a HFMD outbreak in Finland in 2008 was the earliest strain. While, the KM114057(Fin/08) together with the strain isolated in France in 2010 was in a distinct sub-cluster and separated from the predominant circulating strains in China (Fig. 2). These data suggest that the CVA6 strain causing the large HFMD epidemic in 2013 likely originates from local circulating strains in mainland China rather than being imported from other countries.

In other countries such as Japan, Singapore, Taiwan, and Vietnam, cyclical epidemics of HFMD have occurred every 2–3 years^5^,^41^,^42^,^43^. To date, it is still not clear why most regions of China have experienced this continued and even increased HFMD epidemic. However our study provides a possible explanation. EVA71, CVA16 and CVA6 are the three prevalent serotypes of enterovirus that have circulated in Guangdong China in the last six years. In most years, one of the three serotypes have dominated the epidemic activity, representing about 50% enterovirus positive cases i.e.EVA71 in 2008 and 2010, CVA16 in 2009, and CVA6 in 2013. The human population may develop immunity to the predominant serotype and reduce the number of the susceptible population (mainly the children less than 3 years old) to the virus infection. However, the immune response induced by the predominant serotype cannot protect the population from infection of other serotypes of enterovirus^44^. The enterovirus may take advantage of this rotating viral serotytpe pattern to evade from population immunity resulting in the accumulation of susceptible populations and annual HFMD epidemics in China. Further studies are warranted to better elucidate the dynamics and immunity of the population and to provide more clues on the pattern of HFMD epidemics. More importantly, though EVA71 virus vaccines are being developed in China and are at different clinical trial stages now^14^,^15^, considering the pattern of HFMD epidemics described in this study, our current strategies on controlling HFMD should be improved.

In conclusion, the long term epidemiology study of CVA6 reveals that CVA6 has been one of three major causative agents for HFMD epidemics in Guangdong China. By rotating among the EVA71, CVA16 and CVA6 serotypes, the enterovirus appears to evade from large scale population immunity resulting in continuously increasing HFMD epidemics in the past six years in mainland China. In addition, the genetic analysis identifies that the sub-genotype A of CVA6 has replaced the sub-genotype D to become the predominant viral strain in successive epidemics in Guangdong, China. Due to the repeated epidemic of HFMD, ongoing studies are needed to confirm the pattern of the epidemic and to determine virulence of different genotypes of CVA6.

## Material and methods

### HFMD surveillance and sample collection

The Guangdong provincial web-based HFMD surveillance system has been established since 2008 when HFMD was defined as a category C notifiable disease^26^,^45^. All suspected cases were required to be reported to the web-based surveillance system within 24 hours of diagnosis for category C infectious disease. A total of 871 clinics and local Centers for Disease Control (CDC) in 21 cities in Guangdong have joined the HFMD web-based surveillance network. Clinics are responsible for reporting all cases to the local CDC 24 hours after diagnosis. HFMD cases are identified according to the Ministry of Health diagnostic criteria, and the case definition of HFMD includes mild and severe cases based on the official guidance material ( http://www.nhfpc.gov.cn/jkj/index.shtml). The mild cases were defined by the presence or absence of fever with a rash of papula or herpetic lesions on the buttocks. The severe cases were defined as HFMD cases complicated with encephalitis, aseptic meningitis, acute flaccid paralysis, myocarditis, autonomic nervous system dysregulation (ANS), pulmonary edema (PE), pulmonary hemorrhage, severe sequelae or even death^25^.

In the HFMD surveillance system, local CDCs from 21 cities in Guangdong are responsible for sample collection from sentinel hospitals. Stools, throat and rectal swabs collected from suspected cases are first used to detect the presence of enterovirus and then the EVA71 and CVA16 in local CDC laboratories^45^. Positive results are reported to Guangdong provincial CDC with the clinical specimen delivered for further virus isolation and sequencing. Information about the patients including age, sex, and clinical symptoms (mild or severe cases) are available from the provincial web-based surveillance system of HFMD. Besides the HFMD samples, nine strains of CVA6 were detected from samples of acute flaccid paralysis (AFP) cases from 2004 to 2007. Systematic AFP surveillance was initiated in Guangdong in 2000 using the standard approach recommended by World Health Organization (WHO) for polio surveillance ( http://www.who.int/en/).

### Molecular typing and VP1 sequencing

Viral RNA was directly extracted from the collected clinical samples with a QIAamp Viral RNA mini kit (52904, QIAGEN, USA) according to the manufacturer’s instructions. The real-time PCR (RT-PCR) was firstly performed for detecting the presence of the common (universal) sequence of enterovirus, and the specific sequences of EVA71 and CVA16 with enterovirus detection kit (DA-BN165, DAAN Gene, Guangzhou, China). For non-EVA71 and non-CVA16 enterovirus, CVA6-specific real-time RT-PCR was performed by using commercial kit (JC20106, bioPerfectus technologies, Jiangsu, China). For VP1 sequencing, PCR was performed with a QIAGEN OneStep RT-PCR Kit and CVA6 specific primer (nucleotides 2335 to 3420, relative to Coxsackievirus A6 strain Gdula), CA6-VP1-F:5’-TGTGCAAGGACACYGAYGAG-3’ and CA6-VP1-R: 5’-AGATGYCGGTTTACCACTCT-3’. All CVA6 VP1 sequences have been submitted to GenBank (accession no. KM079501-KM079588, KP143073-KP143081).

### Phylogenetic analysis

97 newly sequenced CVA6 together with 69 CVA6 previously sequenced^22^ were aligned with reference sequences using the Clustal W program implemented in Bioedit 7.2^46^. The reference sequences that represented all known CVA6 sub-genotypes were obtained from the GenBank database. A phylogenetic tree was constructed in a maximum likelihood (ML) framework using MEGA version 6.0 with the general time reversible GTR+G+I model and 1000 replicates^47^. The large phylogenetic tree included all CVA6 from GenBank database were firstly constructed. Based on this data, the small phylogenetic tree was further constructed with selected representative taxa.

### Statistical Analysis

Data were analyzed using statistical software SPSS18.0. The statistical differences in the male/female ratio and severe/mild ratio between different enterovirus serotypes were tested by chi-squared test. Analysis of variance was used to compare means by age. A *P-value* < 0.05 was regarded as statistically significant.

## Author Contributions

H.Z., J.L. and H.L. designed the study; H.Z., L.Y., X.G., L.L, L.S. and X.T. performed experiments; C.K., J.L and J.L analyzed data; J.L. and S.R. wrote the manuscript.

## Additional Information

**How to cite this article**: Zeng, H. *et al*. The Epidemiological Study of Coxsackievirus A6 revealing Hand, Foot and Mouth Disease Epidemic patterns in Guangdong, China. *Sci. Rep.* **5**, 10550; doi: 10.1038/srep10550 (2015).

## Supplementary Material

Supplementary Information

## Figures and Tables

**Table 1:** Demographic and clinical features of patients with different enterovirus infection.

**Characteristics**	**CV-A6 (n = 1026)**	**EV71 (n = 1560)**	**CVA16 (n = 997)**
Median Age, months (range)	17, (1-336)	19, (1-478)	20, (1-264)
Sex, male (%)	676 (65.9%)	1011 (64.8%)	635 (63.7%)
Clinical Outcome			
Mild	989 (96.4%)*	1110 (71.2%)	965 (96.7%)*
Severe	37 (3.6%)*	450 (28.8%)	32 (3.3%)*

Significant variations were evaluated by using Chi-squared test.

^*^Compared with EV71 infection group, P < 0.05.
