# Supplementary material for: The Epidemiological Study of Coxsackievirus A6 revealing Hand, Foot and Mouth Disease Epidemic patterns in Guangdong, China
Source: Sci Rep. 2015 May 21;5:10550. doi: 10.1038/srep10550 (PMC4440203; doi:10.1038/srep10550)
Supplement: Supplementary Information [file srep10550-s1.pdf]

The Epidemiological Study of Coxsackievirus A6 revealing Hand, Foot and Mouth Disease Epidemic patterns in Guangdong, China

Hanri Zeng, Jing Lu, Huanying Zheng, Lina Yi, Xue Guo, Leng Liu, Shannon Rutherford, Limei Sun, Xiaohua Tan, Hui Li, Changwen Ke and Jinyan Lin

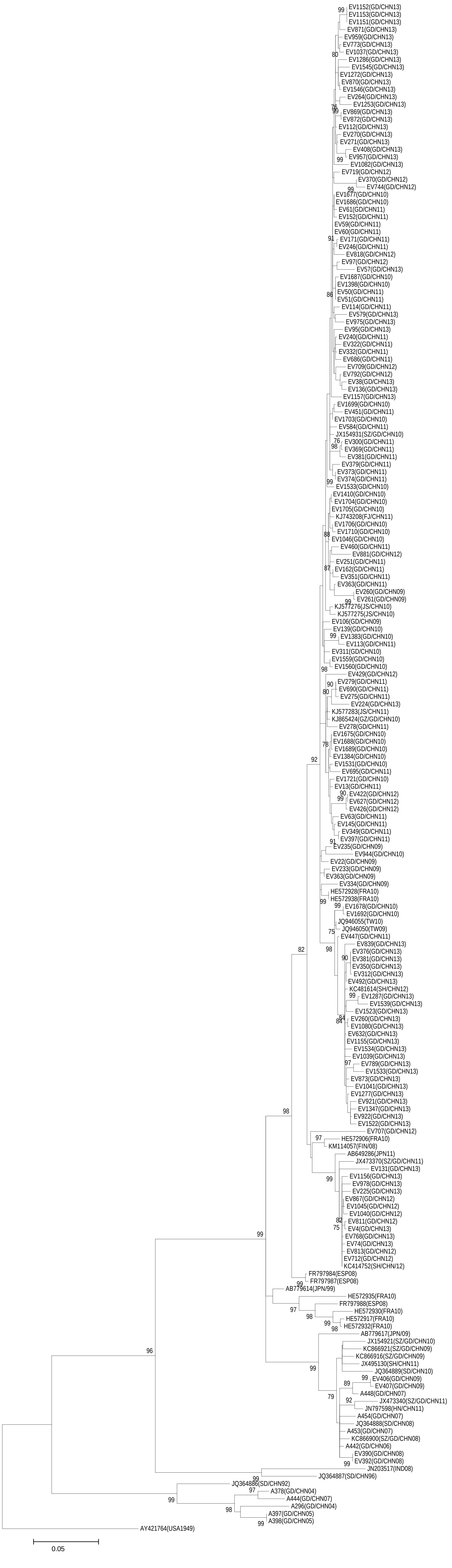
EV1152(GD/CHN13)
EV1153(GD/CHN13)
EV1151(GD/CHN13)
EV871(GD/CHN13)
EV959(GD/CHN13)
EV773(GD/CHN13)
EV1037(GD/CHN13)
EV1286(GD/CHN13)
EV1545(GD/CHN13)
EV1272(GD/CHN13)
EV870(GD/CHN13)
EV1546(GD/CHN13)
EV264(GD/CHN13)
EV1253(GD/CHN13)
EV869(GD/CHN13)
EV872(GD/CHN13)
EV112(GD/CHN13)
EV270(GD/CHN13)
EV271(GD/CHN13)
EV408(GD/CHN13)
EV957(GD/CHN13)
EV1082(GD/CHN13)
EV719(GD/CHN12)
EV370(GD/CHN12)
EV744(GD/CHN12)
EV1677(GD/CHN10)
EV1686(GD/CHN10)
EV61(GD/CHN11)
EV152(GD/CHN11)
EV59(GD/CHN11)
EV60(GD/CHN11)
EV171(GD/CHN11)
EV246(GD/CHN11)
EV818(GD/CHN12)
EV97(GD/CHN12)
EV57(GD/CHN13)
EV1687(GD/CHN10)
EV1398(GD/CHN10)
EV50(GD/CHN11)
EV51(GD/CHN11)
EV114(GD/CHN11)
EV579(GD/CHN13)
EV975(GD/CHN13)
EV95(GD/CHN13)
EV240(GD/CHN11)
EV322(GD/CHN11)
EV332(GD/CHN11)
EV686(GD/CHN11)
EV709(GD/CHN12)
EV792(GD/CHN12)
EV38(GD/CHN13)
EV136(GD/CHN13)
EV1157(GD/CHN13)
EV1699(GD/CHN10)
EV451(GD/CHN11)
EV1703(GD/CHN10)
EV584(GD/CHN11)
JX154931(SZ/GD/CHN10)
EV300(GD/CHN11)
EV369(GD/CHN11)
EV381(GD/CHN11)
EV379(GD/CHN11)
EV373(GD/CHN11)
EV374(GD/CHN11)
EV1533(GD/CHN10)
EV1410(GD/CHN10)
EV1704(GD/CHN10)
EV1705(GD/CHN10)
KJ743208(FJ/CHN11)
EV1706(GD/CHN10)
EV1710(GD/CHN10)
EV1046(GD/CHN10)
EV460(GD/CHN11)
EV881(GD/CHN12)
EV251(GD/CHN11)
EV162(GD/CHN11)
EV351(GD/CHN11)
EV363(GD/CHN11)
EV260(GD/CHN09)
EV261(GD/CHN09)
KJ577276(JS/CHN10)
KJ577275(JS/CHN10)
EV106(GD/CHN09)
EV139(GD/CHN10)
EV1383(GD/CHN10)
EV113(GD/CHN11)
EV311(GD/CHN10)
EV1559(GD/CHN10)
EV1560(GD/CHN10)
EV429(GD/CHN12)
EV279(GD/CHN11)
EV690(GD/CHN11)
EV275(GD/CHN11)
EV224(GD/CHN13)
KJ577283(JS/CHN11)
KJ865424(GZ/GD/CHN10)
EV278(GD/CHN11)
EV1675(GD/CHN10)
EV1688(GD/CHN10)
EV1689(GD/CHN10)
EV1384(GD/CHN10)
EV1531(GD/CHN10)
EV695(GD/CHN11)
EV1721(GD/CHN10)
EV13(GD/CHN11)
EV422(GD/CHN12)
EV627(GD/CHN12)
EV426(GD/CHN12)
EV63(GD/CHN11)
EV145(GD/CHN11)
EV349(GD/CHN11)
EV397(GD/CHN11)
EV235(GD/CHN09)
EV944(GD/CHN10)
EV22(GD/CHN09)
EV233(GD/CHN09)
EV363(GD/CHN09)
EV334(GD/CHN09)
HE572928(FRA10)
HE572938(FRA10)
EV1678(GD/CHN10)
EV1692(GD/CHN10)
JQ946055(TW10)
JQ946050(TW09)
EV447(GD/CHN11)
EV839(GD/CHN13)
EV376(GD/CHN13)
EV381(GD/CHN13)
EV350(GD/CHN13)
EV312(GD/CHN13)
EV492(GD/CHN13)
KC481614(SH/CHN12)
EV1287(GD/CHN13)
EV1539(GD/CHN13)
EV1523(GD/CHN13)
EV260(GD/CHN13)
EV1080(GD/CHN13)
EV632(GD/CHN13)
EV1155(GD/CHN13)
EV1534(GD/CHN13)
EV1039(GD/CHN13)
EV789(GD/CHN13)
EV1533(GD/CHN13)
EV873(GD/CHN13)
EV1041(GD/CHN13)
EV1277(GD/CHN13)
EV921(GD/CHN13)
EV1347(GD/CHN13)
EV922(GD/CHN13)
EV1522(GD/CHN13)
EV707(GD/CHN12)
HE572906(FRA10)
KM114057(FIN/08)
AB649286(JPN11)
JX473370(SZ/GD/CHN11)
EV131(GD/CHN13)
EV1156(GD/CHN13)
EV978(GD/CHN13)
EV225(GD/CHN13)
EV867(GD/CHN12)
EV1045(GD/CHN12)
EV1040(GD/CHN12)
EV811(GD/CHN12)
EV4(GD/CHN13)
EV768(GD/CHN13)
EV74(GD/CHN13)
EV813(GD/CHN12)
EV712(GD/CHN12)
KC414752(SH/CHN/12)
FR797984(ESP08)
FR797987(ESP08)
AB779614(JPN/99)
HE572935(FRA10)
FR797988(ESP08)
HE572930(FRA10)
HE572917(FRA10)
HE572932(FRA10)
AB779617(JPN/09)
JX154921(SZ/GD/CHN10)
KC866921(SZ/GD/CHN09)
KC866916(SZ/GD/CHN09)
JX495130(SH/CHN11)
JQ364889(SD/CHN10)
EV406(GD/CHN09)
EV407(GD/CHN09)
A448(GD/CHN07)
JX473340(SZ/GD/CHN11)
JN797598(HN/CHN11)
A454(GD/CHN07)
JQ364888(SD/CHN08)
A453(GD/CHN07)
KC866900(SZ/GD/CHN08)
A442(GD/CHN06)
EV390(GD/CHN08)
EV392(GD/CHN08)
JN203517(IND08)
JQ364887(SD/CHN96)
JQ364886(SD/CHN92)
A378(GD/CHN04)
A444(GD/CHN07)
A296(GD/CHN04)
A397(GD/CHN05)
A398(GD/CHN05)
AY421764(USA1949)
0.05

Fig. S1. Phylogenetic analysis of coxsackievirus A6 viral capsid protein 1 nucleotide sequences (nt 2441 to 3355 according to Gdula strain AY421764) showing the relationships between CVA6 strains. A total of 166 CVA6 collected in Guangdong from 2004 to 2013 were included. Scale bar indicates branch distances; solid circles indicate strains sequenced in this study. Only bootstrap values of over 75% are shown.
